# Technological Innovations in Disease Management: Text Mining US Patent Data From 1995 to 2017

**DOI:** 10.2196/13316

**Published:** 2019-04-30

**Authors:** Ming Huang, Maryam Zolnoori, Joyce E Balls-Berry, Tabetha A Brockman, Christi A Patten, Lixia Yao

**Affiliations:** 1 Department of Health Sciences Research Mayo Clinic Rochester, MN United States; 2 Center for Clinical and Translational Science Commuity Engagement Program Mayo Clinic Rochester, MN United States; 3 Department of Psychiatry and Psychology Mayo Clinic Rochester, MN United States

**Keywords:** patent, technological innovation, disease, research opportunity index, public health index, text mining, topic modeling, dynamic topic model, resource allocation, research priority

## Abstract

**Background:**

Patents are important intellectual property protecting technological innovations that inspire efficient research and development in biomedicine. The number of awarded patents serves as an important indicator of economic growth and technological innovation. Researchers have mined patents to characterize the focuses and trends of technological innovations in many fields.

**Objective:**

To expand patent mining to biomedicine and facilitate future resource allocation in biomedical research for the United States, we analyzed US patent documents to determine the focuses and trends of protected technological innovations across the entire disease landscape.

**Methods:**

We analyzed more than 5 million US patent documents between 1995 and 2017, using summary statistics and dynamic topic modeling. More specifically, we investigated the disease coverage and latent topics in patent documents over time. We also incorporated the patent data into the calculation of our recently developed Research Opportunity Index (ROI) and Public Health Index (PHI), to recalibrate the resource allocation in biomedical research.

**Results:**

Our analysis showed that protected technological innovations have been primarily focused on socioeconomically critical diseases such as “other cancers” (malignant neoplasm of head, face, neck, abdomen, pelvis, or limb; disseminated malignant neoplasm; Merkel cell carcinoma; and malignant neoplasm, malignant carcinoid tumors, neuroendocrine tumor, and carcinoma in situ of an unspecified site), diabetes mellitus, and obesity. The United States has significantly improved resource allocation to biomedical research and development over the past 17 years, as illustrated by the decreasing PHI. Diseases with positive ROI, such as ankle and foot fracture, indicate potential research opportunities for the future. Development of novel chemical or biological drugs and electrical devices for diagnosis and disease management is the dominating topic in patented inventions.

**Conclusions:**

This multifaceted analysis of patent documents provides a deep understanding of the focuses and trends of technological innovations in disease management in patents. Our findings offer insights into future research and innovation opportunities and provide actionable information to facilitate policy makers, payers, and investors to make better evidence-based decisions regarding resource allocation in biomedicine.

## Introduction

Patents are an important form of intellectual property that grants inventors monopolies for a limited period of time and provides inventors with a financial incentive for commercialization. Without such financial incentive, private investors in the pharmaceutical and medical device industries may be reluctant to invest in new technologies, which would then slow down the development of new diagnoses and treatments [1]. As patents can promote economically efficient research and development, the number of patents has been used as a proxy for technological innovation and an indicator of economic growth [2]. Patent documents describe the inventor, owner, abstract, claims, and legal status of patented inventions and are publicly available. They have been mined to identify focuses and trends of technological innovations in many areas such as the fisheries sector [3], solar cell industry [4], and drug discovery [5]. A comprehensive survey paper is available to gain a better understanding of the structure of patent documents and the methods for patent document retrieval, classification, and visualization [6].

Existing patent mining in biomedicine mostly focuses on recognizing biomedical entities such as chemical compounds, genes, proteins, cells, tissues, and anatomical parts [7]. For example, Leman et al developed a system of named entity recognition to identify chemical names mentioned in the patents [8]. Fechete et al mined all gene names mentioned in the claim section of diabetic nephropathy-related patents [9]. Grouin applied machine learning approaches to detect pharmacological terms such as target population, organs, symptoms, and treatments [10]. Information mined from patents has been further used to formulate new biomedical hypotheses [9] and discover technological trends about treatment of a specific disease [11]. For instance, Gwak et al identified the trends and leading organizations in wound-healing technology [11] for successful investment strategies and policy making in the future.

One missing aspect in biomedical patent mining is identification of focuses and trends of patented inventions across the entire disease landscape, in order to facilitate evidence-based decision making for future resource allocation in biomedical research. Therefore, in this study, we mined US patent documents from 1995 to 2017 to identify the trends of patent coverage for over 600 diseases and medical conditions. We then incorporated patent coverage for diseases and medical conditions to recalibrate the Research Opportunity Index (ROI) and Public Health Index (PHI) in order to systematically understand resource allocation and research prioritization. ROI and PHI were introduced in our previous work [12] to measure the (im)balance between the health burden associated with a particular disease or medical condition or all diseases and medical conditions as a whole, and the allocated resources. Previously, we used treatment cost as proxies of disease burden and the numbers of scientific publications and clinical trials as indicators of resource allocations. By incorporating patent documents, we considered technological innovation as a driver of resource allocation and research prioritization in biomedicine, which impacts the entire biomedical research ecosystem. Finally, we performed dynamic topic modeling [13] to uncover the latent topics of patented inventions associated with each disease or medical condition and the trend of these topics over time. This study could provide insights into research and development opportunities and offer actionable information for future investment and funding decision making in biomedicine.

## Methods

**Figure 1:**
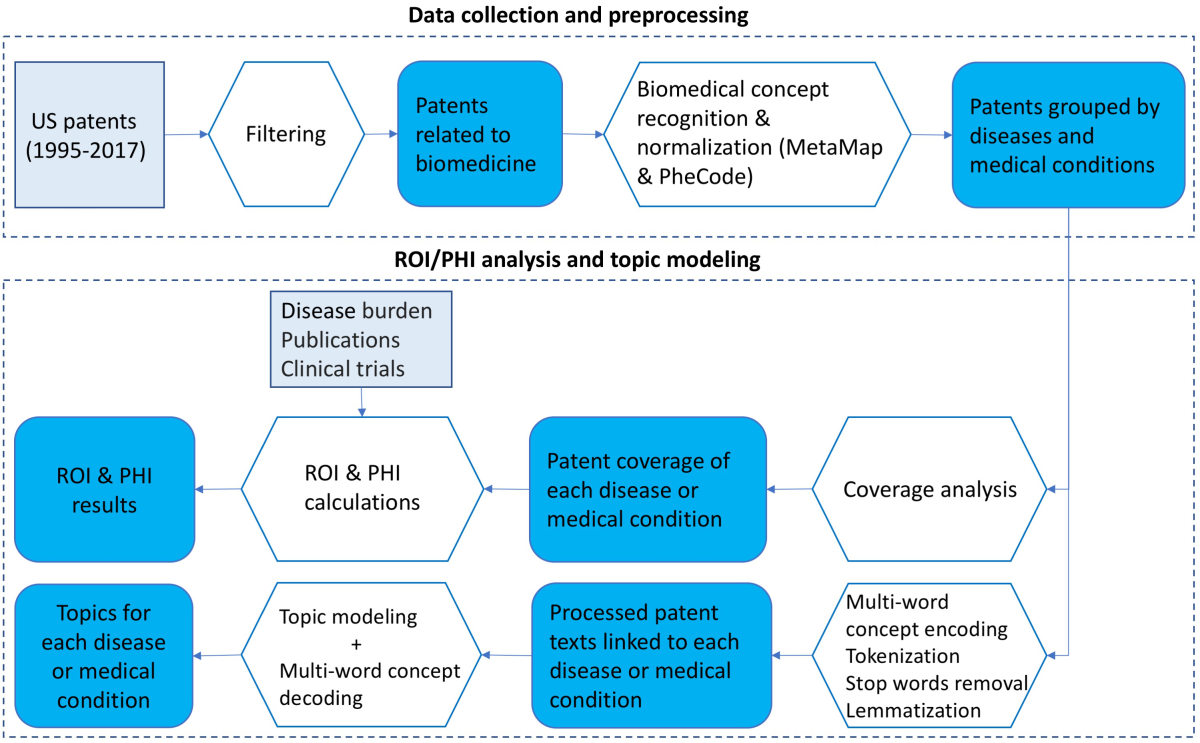
Study workflow of mining US patent data. PHI: Public Health Index; ROI: Research Opportunity Index.

The workflow of this study is illustrated in Figure 1. It includes two phases: (1) data collection and preprocessing and (2) ROI/PHI analysis and topic modeling. Below, we describe each step in more detail.

### Patent Data Collection and Filtering

We downloaded approved US patents between 1995 and 2017 from the US Patent and Trademark Office website [14]. The whole dataset included more than 5 million patent documents, whose formats changed three times: Green Book format (1995-2001) [15], Red Book Standard Generalized Markup Language (SGML; 2002-2004) [16], and Red Book XML (2005-2017) [17]. These patent documents were classified into different innovative domains (eg, agriculture, sports, and foodstuffs) based on the United States Patent Classification (USPC) system [18] and the Cooperative Patent Classification (CPC) system [19]. We set the following inclusion criteria to extract patent documents on biomedicine: Publication date should be between January 1, 1995, and December 31, 2017, and the patent document should contain at least one USPC or CPC classification code listed in Multimedia Appendix 1.

Any documents that did not meet the abovementioned criteria were not considered to be related to biomedicine. We developed a python parser (Multimedia Appendix 2) to parse patent documents in all three formats in order to retrieve information such as patent identification, issue date, title, abstract, claims, and USPC and CPC classification codes. We then filtered for biomedicine-related patents using the compiled lists of USPC and CPC classification codes (Multimedia Appendix 1).

### Biomedical Concept Recognition and Normalization

For patent documents related to biomedicine, we used MetaMap, an application developed at the National Library of Medicine [20] to extract and map biomedical concepts (key words or phrases) of 74 semantic types in the sections of title, abstract, and claims to the Unified Medical Language System (UMLS) metathesaurus. The complete list of 74 UMLS semantic types is shown in Multimedia Appendix 1.

UMLS assigns a Concept Unique Identifier (CUI) to each concept and links it to the source thesauri such as the International Classification of Diseases, Ninth Revision, Clinical Modification (ICD-9-CM) [21]. We leveraged ICD-9-CM to identify concepts of diseases and medical conditions in patent documents and mapped ICD-9-CM to PheCode, which represents clinically meaningful phenotypes used by clinicians [22]. To address the issue of concept granularity, we only used the 704 root PheCodes such as diabetes mellitus, influenza, and pain. Thus, patent documents were grouped by diseases and medical conditions before ROI/PHI and topic modeling analysis.

### Coverage Analysis

In the second phase, we started by calculating the patent coverage of each disease or medical condition by dividing the number of patent documents mentioning the disease or medical condition by the total number of patent documents mentioning all diseases and medical conditions in each year. This is a preparation step for computing the ROI for each disease or medical condition and PHI for all diseases and medical conditions in each year.

### Multiword Concept Encoding, Tokenization, Stop Word Removal, and Lemmatization

To prepare for topic modeling, we encoded multiword biomedical concepts using their corresponding CUIs before tokenizing the patent documents, removing stop words, and lemmatizing words. Multiword concept encoding helps preserve compound concepts such as “type 2 diabetes.” Without multiword concept encoding, such a compound name would be broken down to individual words (unigram) during topic modeling. Tokenization breaks text into smaller meaningful elements such as words, numbers, or punctuation marks. Stop words like “the,” “is,” and “are” are usually filtered out from natural language processing. Lemmatization aims to reduce the morphological variations of words by returning to the base or dictionary form of a word (eg, “walked,” “walking,” and “walks” have the same base form “walk”).

### Data on Disease Burden, Publications, and Clinical Trials

The data on disease burden, publications, and clinical trials were collected in the same way as that in our previous work [12]. However, in this study, we included more data from recent years to obtain an updated longitudinal analysis for 17 years (2000-2016). Disease burden was estimated using the total treatment cost in million population each year from OptumLabs Data Warehouse [23]. OptumLabs Data Warehouse is a comprehensive, de-identified administrative claims database for commercially insured and Medicare Advantage enrollees in a large and private US health plan. The diagnosis records were coded by ICD-9-CM before October 2015 and International Classification of Diseases, Tenth Revision, Clinical Modification (ICD-10-CM) [24] after those claims.

For publications, we downloaded all Medical Subject Heading (MeSH)-indexed abstracts in English from the MEDLINE database (via the PubMed query interface), the biomedical publication database maintained by the United States National Library of Medicine from 2000 to 2016. Subsequently, we used the number of publications annotated by each MeSH disease or medical condition term to approximate the attention it received from the biomedical research community. We also downloaded the aggregated, MeSH-indexed clinical trial database [25]. Similarly, we used the number of clinical trials related to each specific disease or medical condition to approximate feasibility and popularity of carrying out clinical research in each disease or medical condition area. We converted ICD-9-CM, ICD-10-CM, and MeSH codes to the root PheCode before further analysis.

### Research Opportunity Index and Public Health Index Calculations

We previously proposed ROI and PHI for quantitatively measuring resource allocation for a particular disease and all the medical conditions as a whole [12]. The calculations of ROI and PHI are flexible and can incorporate many quantitative factors that impact research prioritization and resource allocation in biomedicine. In this work, we updated the ROI and PHI calculations by including more data from recent years and included patent data in addition to disease burden, publications, and clinical trials, since patents are an important form of intellectual property on technological innovations.

ROI and PHI are defined as follows: 
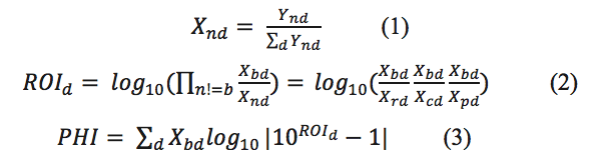


where *Y*_*nd*_ is the raw measurement *n* for a disease *d.* In our model, we used treatment cost per million people as indicators of burden of disease (*Y*_*bd*_) and the number of research publications (*Y*_*rd*_), the number of clinical trials (*Y*_*cd*_), and the number patent documents (*Y*_*pd*_) as an approximation of resources spent in biomedical research and development. Since the raw measurement *Y*_*nd*_ is subjective to inflation (eg, increasing treatment cost and number of publications over time) and cannot be compared across different units (eg, treatment cost in dollars vs number of research publications by count), we used the normalized measure *X*_*nd*_ instead of *Y*_*nd*_ in ROI and PHI calculation. ROI quantifies the imbalance between needs and resource investment on multiple dimensions for each disease or medical condition. PHI describes the overall resource allocation efficiency for all diseases and medical conditions.

### Topic Modeling

Topic modeling automatically uncovers the topics or themes in a large collection of documents, in terms of a set of keywords occurring together and most frequently [26-29]. We applied a dynamic topic model (DTM) [13] to learn the topics in patent documents related to specific diseases and medical conditions and the evolution of these topics over years.

DTM is an extension of the static Latent Dirichlet Allocation method [26] for analyzing the temporal changes of topics of a large collection of documents. Static Latent Dirichlet Allocation does not consider the input order of documents in the large collection. It assumes the Dirichlet prior distributions for topic distributions in a document and word distributions over a topic. DTM recognizes the importance of temporality in a large collection of documents and studies the dynamics of topics from time interval *t-1* to *t.* It assumes a Gaussian distribution for the prior parameters at *t*, given their value at *t-1*.

We used the open-source DTM C++ package [30] wrapped in Gensim library [31] to learn the temporality of the topics in patent documents related to a specific disease or medical condition. After multiword concept encoding, tokenization, stop word removal, and lemmatization, each patent document was converted into a vocabulary vector, where the elements were frequency of each lemma (including CUIs) without considering the order of lemma. As such, all the patent documents on the same disease or medical condition were converted into a *D* × *V* matrix, where *D* stands for count of patent documents and *V* denotes the size of the entire vocabulary in those patent documents. The *D* × *V* matrix was then chunked into time intervals in the calendar year for dynamic topic modelling.

We calculated topic coherence [32] quantitatively and asked domain experts to qualitatively evaluate the learned topics. More specifically, we evaluated the topic coherence at different topic numbers (ie, 2, 4, 6, 8, 10, 12, 14, 16, 18, and 20) to determine the optimal topic number for three selected diseases: diabetes mellitus, breast cancer, and epilepsy. We found that the optimal topic number was 6 for breast cancer–related patents, 8 for diabetes mellitus–related patents, and 16 for epilepsy-related patents (Multimedia Appendix 1). The average number of topics for the three diseases was 10. Thus, we set the topic number to 10 empirically for the remaining 639 identified diseases and medical conditions.

We also investigated the hyperparameters of *α* and *σ* in DTM: *α* controls the topic distributions over a document, and a smaller *α* results in fewer topics statistically associated with a document, whereas *σ* determines how fast the topics evolve over time, and a smaller *σ* leads to more similar word distributions over a topic over time. In our experiment, we used the default values of 0.01 and 0.005 for *α* and *σ*, respectively, as previously suggested [33], as those authors reported that both α and σ did not affect topic distributions and word distributions significantly over time and did not have an effect on topic interpretation by domain experts.

## Results

### Disease Coverage in Patent Documents During 1995-2017

**Figure 2:**
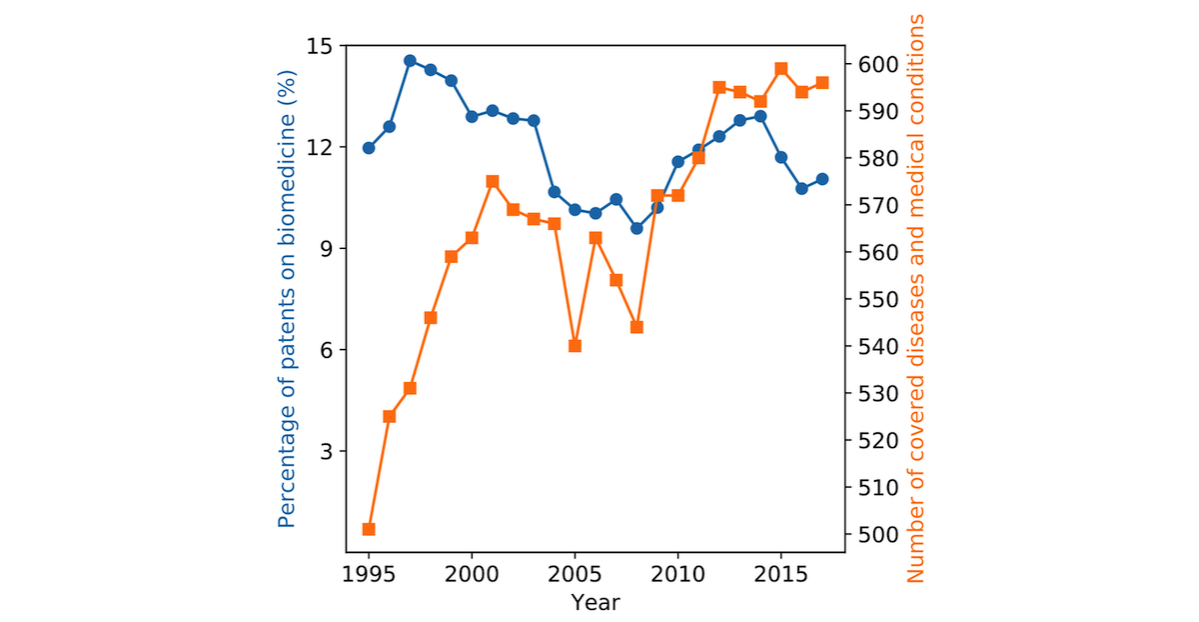
The percentage of patent documents related to biomedicine (blue dots) and the number of diseases and medical conditions covered in patent documents (orange squares) during 1995-2017.

We collected 5,010,329 patent documents from 1995 to 2017, of which 550,961 (about 11%) were related to biomedicine. Figure 2 shows the percentage of patent documents related to biomedicine and the number of diseases and medical conditions covered in those patent documents in each year. It seemed that the approved US patents on biomedicine fluctuated in the range of 9.6%-14.6% during 1995 and 2017. However, the number of diseases and medical conditions covered in US patents expanded from 502 to 596, suggesting that technology innovations had been focusing on more diseases and medical conditions during the same time.

**Figure 3:**
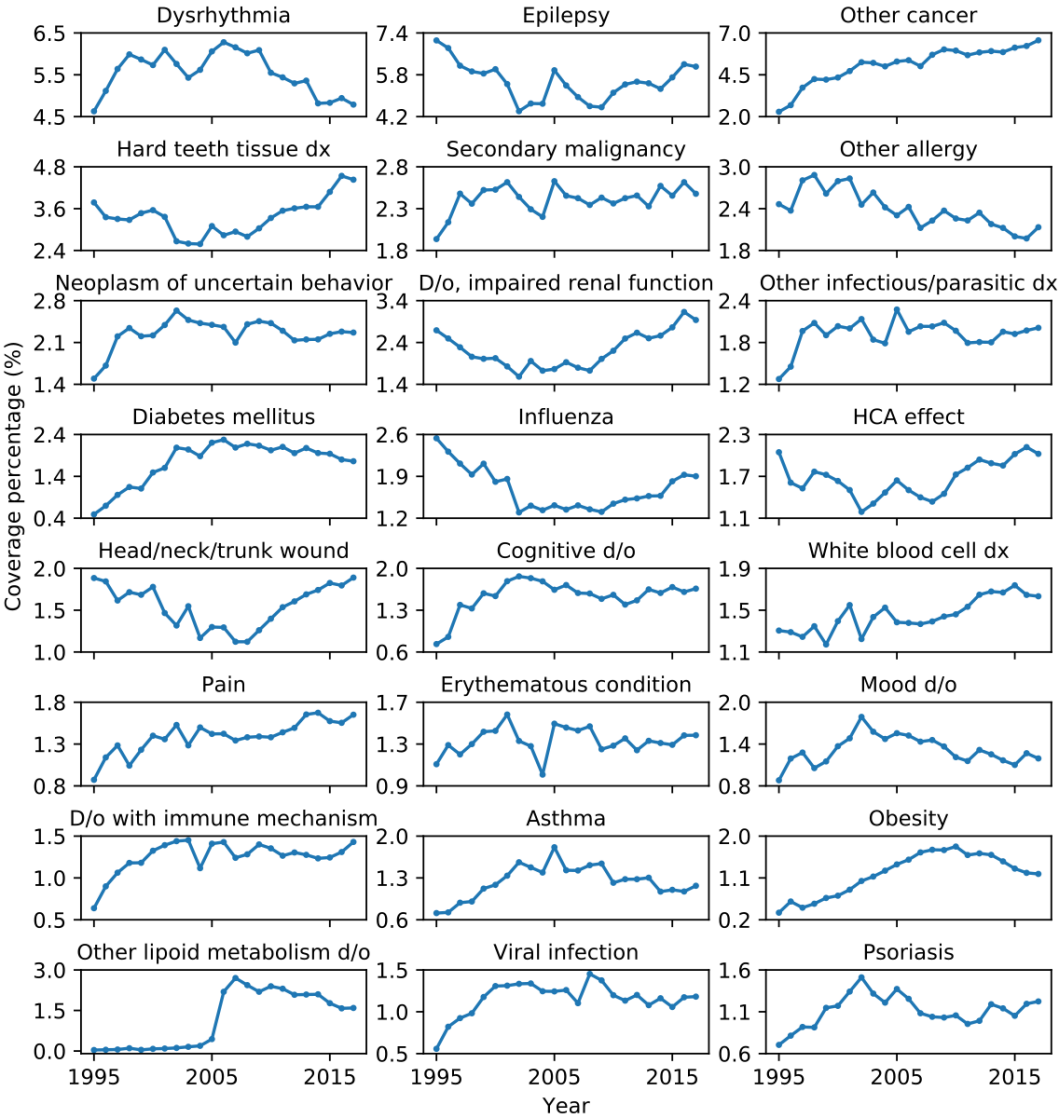
Patent coverage of top 24 mentioned diseases and medical conditions during 1995-2017. Cognitive d/o refers to delirium, dementia, amnesia, and other cognitive disorders. Obesity refers to overweight, obesity, and other hyperalimentation. dx: disease; d/o: disorder; HCA: heat, cold, and air pressure.

Figure 3 shows the coverage percentages of 24 most mentioned diseases and medical conditions in US patent documents between 1995 and 2017. We found that 16 of them were chronic conditions such as dysrhythmia, “other cancer,” diabetes mellitus, and obesity. “Other cancer” refers to malignant neoplasm of head, face, neck, abdomen, pelvis, or limb; disseminated malignant neoplasm; Merkel cell carcinoma; and malignant neoplasm, malignant carcinoid tumors, neuroendocrine tumor, and carcinoma in situ of an unspecified site, according to PheCode [22]. The percentages of patented inventions on “other cancer” are shown to be steadily increasing (from 2.29% in 1995 to 6.56% in 2017). This time period witnessed a steady increase of innovative technologies from functional magnetic resonance imaging to immunotherapy, which improve cancer diagnosis and treatment [34]. With increasing awareness and expenditure on cancer diagnosis and treatment [35], we believe that technological innovations associated with cancer are likely to grow continuously. Similarly, obesity ranked 21st in the most mentioned 24 diseases and medical conditions, and its patent coverage ranged from 0.87% to 1.65% during the study period. Such an increase in patented inventions for obesity aligns well with its high prevalence in the US, the severe implications on people’s life and the society, and national strategic plans to deal with the obesity epidemic [36]. Influenza, an acute disease, received decreasing attention in terms of patents before 2000 and increasing attention after 2010, reflecting its high prevalence and substantial burden (eg, large morbidity and mortality) on the health care system and society [37]. The Centers for Disease Control and Prevention reported that influenza caused 9.3-49.0 million illnesses, 140,000-960,000 hospitalizations, and 12,000-79,000 deaths in the United States each year since 2010 [38].

### Research Opportunity Index and Public Health Index Analysis

Before ROI and PHI analysis, we examined the multicollinearity between the relative number of patent documents and other factors, namely, the relative treatment costs, the relative number of scientific publications, and the relative number of clinic trials used in our previous model [12]. The low variance inflation factors of 1.0-2.6 (Multimedia Appendix 1) indicated a low multicollinearity between the relative number of patent documents and the other factors [39].

**Figure 4:**
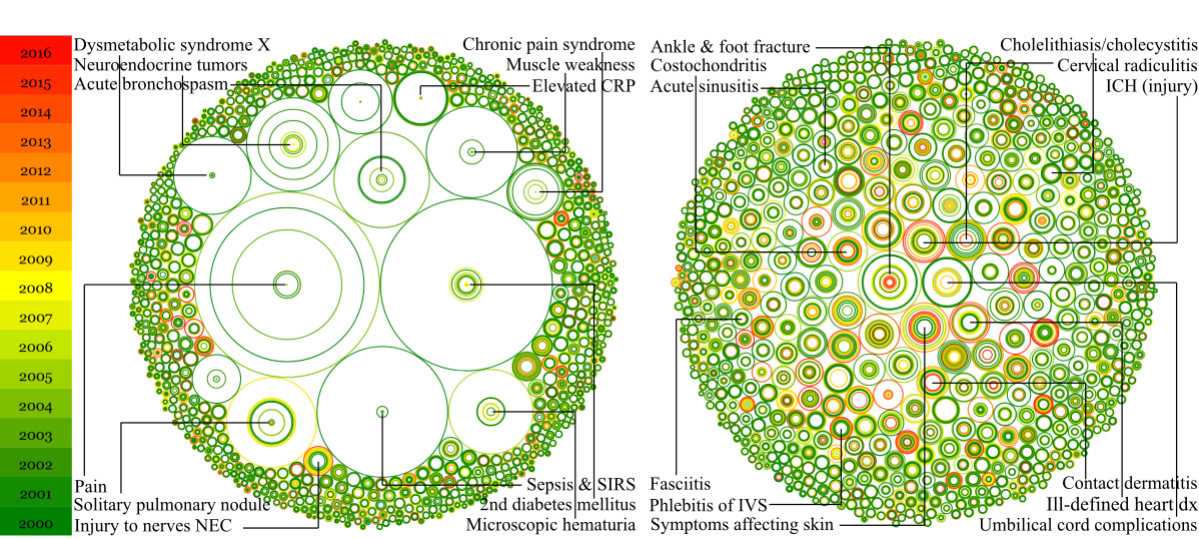
Research Opportunity Index visualization for overstudied (A) and understudied (B) diseases and medical conditions using co-centric circles. A different color of a circle corresponds to a different year, illustrated by the legend on the left. (A) The size of a circle enlarges as the negative Research Opportunity Index of the corresponding disease or medical condition decreases. In other words, the bigger the circle is, the more overstudied is the disease or medical condition. (B) The size of each circle enlarges as the positive Research Opportunity Index of the disease or medical condition increases. In other words, the bigger the circle is, the more understudied is the disease or medical condition, indicating a future research opportunity. Contact dermatitis refers to contact dermatitis and other eczema due to plants except food. dx: disease; NEC: not elsewhere classified; CRP: C-reactive protein; SIRS: systemic inflammatory response syndrome; IVS: intracranial venous sinuses; ICH: intracranial hemorrhage.

We then calculated the ROIs to measure the misalignment between the resources allocated to a disease or medical condition and the burden it imposed between 2000 and 2016, as shown in Figure 4 (see Multimedia Appendix 3 for the raw data). For example, neuroendocrine tumors were overstudied, indicated by negative ROIs from 2000 to 2016. However, their ROI increased from –17.93 (in 2000) to –1.87 (in 2016) over 17 years, implying that resources allocated to it were more aligned with its burden. Further, digging into the dependent variables of ROI, we found that the driving factor for such improvement was that the relative treatment cost (indicator of disease burden) increased much faster than relative publications, relative clinical trials, and relative technological innovations. Similar patterns were observed for most of the overstudied diseases and medical conditions. There were only a few diseases and medical conditions that were more overstudied over time. Injury to nerves not elsewhere classified was such an example. Its ROI scores declined from –6.73 to –9.43 from 2000 to 2016, primarily because its relative treatment cost decreased while it received steady attention in biomedical research (in terms of the relative number of publications) and increasing attention in development (in terms of the relative numbers of clinical trials and patents).

Figure 4 B highlights the understudied diseases and medical conditions with positive ROIs. For example, ankle and foot fracture had steadily declining ROIs (from 12.52 to 6.41) from 2000 to 2016, mostly because the resources allocated to it increased, but its burden slightly decreased over time. More specifically, the relative number of publications increased more than 180 times, the relative number of clinical trials increased over 2 times, and the relative treatment cost decreased by about 20%. The relative numbers of publications, clinical trials, and patents were all disproportionally small, compared to the burden of ankle and foot fracture. In contrast, intracranial hemorrhage (injury) had gradually increasing ROIs (from 6.31 in 2000 to 10.19 in 2016) and was becoming more understudied due to increase in the relative treatment cost and decline in the relative number of publications. There were also diseases and medical conditions that showed fluctuating ROIs over time. For instance, the ROIs for contact dermatitis increased from 6.90 to 8.20 during 2000-2014 and decreased to 5.63 in 2016. Our calculation showed that its relative treatment cost declined steadily by a factor of 1.6 from 2000 to 2016, but its relative number of publications and patents fluctuated dramatically during the same period.

**Figure 5:**
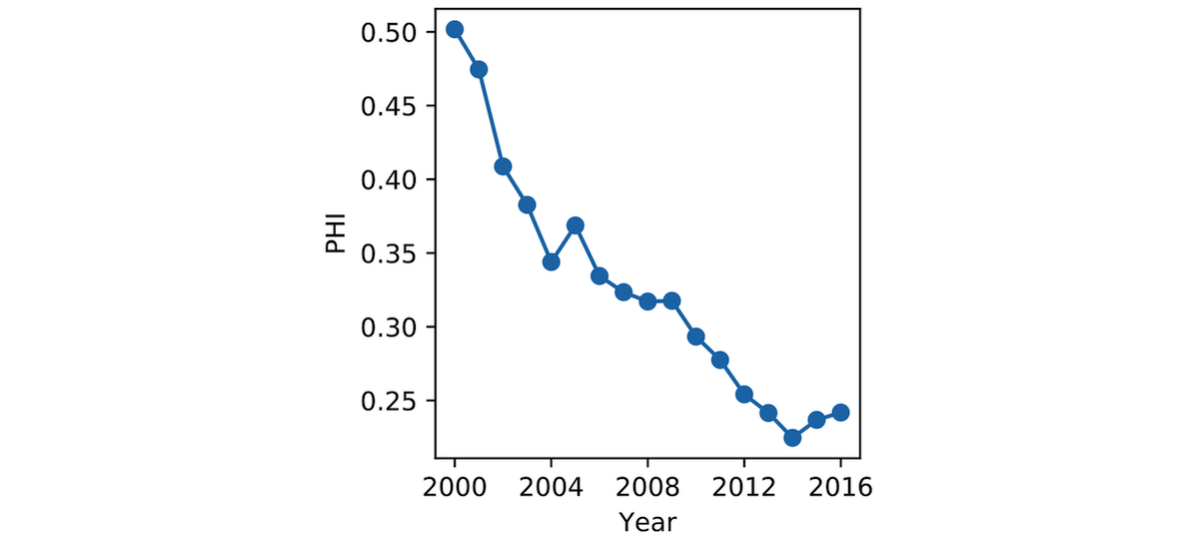
Public Health Index (PHI) during 2000-2016.

The overall alignment between disease burden and allocated resources for all the diseases and medical conditions measured by PHI from 2000 to 2016 is shown in Figure 5. It demonstrated a clear shrinking pattern over the 17-year period, with small increases in 2015 and 2016. As the smaller PHI indicates better alignment between research and development and the distributions of needs across all diseases and medical conditions, the results suggest that the resource allocation for all the diseases and medical conditions as a whole had improved significantly over time.

### Topic Modeling

**Figure 6:**
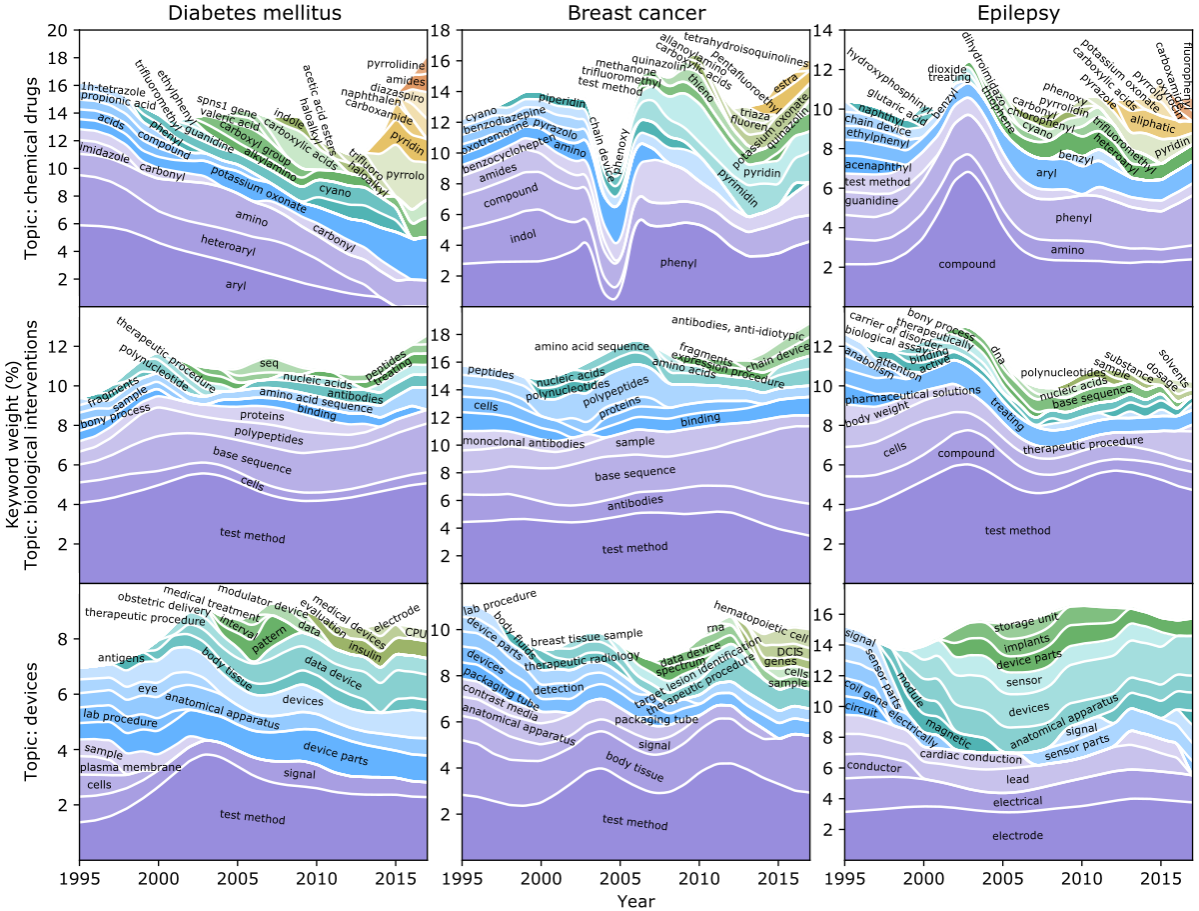
Three meaningful topics identified from patent documents related to diabetes mellitus, breast cancer, and epilepsy, and their changing patterns from 1995 to 2017.

Using a dynamic topic modeling technique, we identified the latent topics and their changing patterns over time for patent documents related to various diseases and medical conditions. In Figure 6, we highlight three meaningful topics identified from patent documents related to diabetes mellitus, breast cancer, and epilepsy, together with their changing patterns from 1995 to 2017. The results for the other diseases and medical conditions are provided in Multimedia Appendix 3.

#### Diabetes Mellitus

With a prevalence of 9.4% among the US population and a financial burden of $245 billion, diabetes mellitus was the seventh leading cause of death in the US in 2015 [40]. Both public and private sectors in the health care industry have invested heavily in developing new methods of diagnosis and treatment for diabetes mellitus. Our dynamic topic modeling analysis showed that most of the patent activities revolved around the development of new drugs (both chemical compounds and biological therapeutics) and new devices to enhance glucose monitoring and diabetes management. More specifically, keywords including “aryl,” “heteroaryl,” and “phenyl” indicate that chemical drugs are a main focus of those patent documents [41]. The top 10 topic keywords also evolved over time; for example, “propionic acid” only appeared during 1995-2000 [42], “valeric acid” appeared during 2004-2006 [43], “indole” appeared during 2008-2009 [43,44], “naphthalen” appeared in 2015 [45], and “diazaspiro” appeared during 2016-2017 [46]. These keywords highlighted the significance of different chemical compounds or functional groups in the development of antidiabetic drugs over years. The topic keywords “base sequence,” “polypeptides,” “antibodies,” and “therapeutic procedure” revealed that biological interventions, gene therapy [47], peptide therapy [48], and immunotherapy [49] were other focuses of the patented innovations. After tracing back to the original patent documents, we found that the identified keywords “signal,” “device parts,” and “devices” were related to devices for improving monitoring [50] and management [51] of glucose level.

#### Breast Cancer

Breast cancer is the most common cancer worldwide [52] and has gained a lot of attention for innovations on diagnosis and therapeutic treatment. Its 5-year relative survival rate increased from 75% in 1975-1977 to 91% in 2006-2012 [53]. The focus of patented inventions related to breast cancer was primarily on the development of novel pharmaceutical drugs, biological products, and devices for diagnosis and treatment. Chemical agents or functional groups such as “phenyl” (1995-2017) [54], “pyrazolo” (1995-2003) [55], “thieno” (2008-2013) [56], and “tetrahydroisoquinolines” (2017) [57] were most mentioned in the top 10 topic words and were reported to have antitumor effects in different years. In addition, “antibodies,” “binding,” “base sequence,” “polypeptides,” and “amino acid sequence” were the most frequent keywords of breast cancer mentioned in patents related to antibody therapy [58] and genetic diagnosis and treatment [59,60]. The keywords on antibody such as “monoclonal antibodies” (1995-2003) and “antibodies, anti-idiotypic” (2014-2017) further reflect the development of patented inventions on antibody therapy for breast cancer [61,62]. The topic words “body tissue,” “signal,” “contrast media,” “devices,” “detection,” “device parts,” “therapeutic radiology,” and “therapeutic procedure” are associated with devices for the detection and treatment for breast cancer [52,63].

#### Epilepsy

Epilepsy is one of the most common neurologic diseases involving about 50 million people worldwide and 3 million people in the United States [64]. It is a chronic disorder that usually consists of unpredictable recurrent seizures with substantial impacts on patients’ mental and physical functioning. Important topics in epilepsy-related patent documents were relevant therapeutic drugs, biological interventions, and electrical devices for epilepsy diagnosis and treatment. During the study period, the patent topic on chemicals involved different keywords such as “phenyl” (1995-2017) [65,66], “naphthyl” (1995-1999) [67], “thiophene” (2004-2005) [68], “pyrrolidin” (2010) [69], and “carboxamide” (2016-2017) [70]. These keywords suggested that these chemical compounds or functional groups were the leading efforts in antiepileptic drug development over years. The topic words “cells,” “therapeutic procedure,” “biological assay,” “base sequence,” “nucleic acids,” and “polynucleotides” disclose patent inventions on biological interventions and gene therapy [71,72]. The keywords “electrode,” “signal,” “devices,” “sensor,” and “implants” suggested that electrical devices for epilepsy diagnosis and intervention were also the focus of patented inventions [73-75].

## Discussion

In this study, we identified 550,961 biomedicine-related patent documents; calculated patent coverage, ROI, and PHI; and performed topic modeling analysis for more than 600 diseases and medical conditions from about two decades. We found that technological innovations reached an increasing number of diseases and medical conditions from 1995 to 2017. The innovation hotspots were around common chronic conditions including “other cancer,” diabetes mellitus, and obesity, which bore significant socioeconomic burden [76]. Technological inventions related to acute conditions, such as influenza, also attained substantial attention due to their high morbidity and mortality [37]. Unfortunately, patents, as a financial incentive to intellectual properties, have not penetrated into many rare diseases yet.

Calculation of PHI from 2000 to 2016 clearly demonstrated that the resource allocation for all the diseases and medical conditions as a whole had improved significantly over time. This is consistent with our previous findings [12], suggesting that the overall resource allocation in biomedical research and development has been improving significantly in the United States, possibly due to more available quantitative data from epidemiology studies and improved transparency in biomedical research and development. Disease-specific ROI tells us whether the resources allocated to a disease align with its burden imposed on the society. The skewness between allocated resources and disease burden measured by treatment cost improved for most overstudied diseases and medical conditions from 2000 to 2016, which possibly contributed to the overall improvement of PHI for all diseases and medical conditions. A few diseases and medical conditions became more overstudied, which demonstrated substantial “inertia” in allocated resources including publications, clinical trials, and patents and their disconnection with disease burden from previous years. One possible explanation is that there was no feedback mechanism to realign resource allocation with disease burden or that the relationship is complex and mediated by other observed variables. For example, researchers’ attention is influenced by exposure to health problems that appear in their local hospitals and clinics, and they have to maintain a relatively stable disease focus for funding and publication purposes in their research career. Negative feedback between patents, clinical trials, scientific studies, and disease burden exists, but operates on a longer timescale than we were able to observe in this study. Diseases such as ankle and foot fracture and intracranial hemorrhage (injury) received positive ROI and thus form a niche for future research and development opportunity.

Additional topic modeling expectedly showed that technological innovations largely focused on developing new diagnosis and treatment for most common chronic and acute diseases and medical conditions, which is in line with several qualitative studies from manual analysis of patent documents [77-79]. The evolution of topic keywords reflects technological development (eg, chemical drugs) on diseases and medical conditions over years.

This study has several limitations. First, disease is not a properly defined concept. Here, we used “diseases and medical conditions” to refer to diseases, syndromes, disorders, symptoms, and abnormalities, as long as they are treated by providers and included in the PheCode taxonomy. No existing medical taxonomy is able to address the issues of granularity, disease comorbidity, and association and the distinction between diseases and symptoms perfectly. However, such imperfection does not revoke the significance of this work, because we are addressing a macroeconomic problem of resource allocation and optimization in biomedicine. Second, there are cases that name variations of biomedical concepts were not listed in the UMLS metathesaurus or MetaMap failed to recognize a disease name and map it correctly to the UMLS metathesaurus [80]. Third, we grouped the recognized CUI for diseases and medical conditions to the root PheCode via ICD-9-CM [21] using mapping tables provided by UMLS and the PheCode team. The percentage of definitive mappings (eg, one to one and multiple to one) from CUI to PheCode is 98.4%, which suggests that the upper bound of error caused by ambiguous mappings (eg, one to multiple or multiple to multiple mappings) might be 1.6%. In addition, in ROI and PHI analysis, we converted ICD-10-CM used in claims database to the root PheCode using ICD-9-CM as a middle layer, as claims database switched from ICD-9-CM to ICD-10-CM in 2015 for coding diseases and medical conditions. We also mapped MeSH terms used by publications and clinical trials to the root PheCode. Imperfectness in the mapping between disease taxonomies could lead to result inaccuracy and interpretation difficulty. Fourth, we used the treatment costs estimated from a large claims database to approximate burdens of diseases and medical conditions when computing the ROI and PHI. We acknowledge that such approximation is far from being perfect because information about uninsured people and uncovered ailments were missing, and human suffering from each disease or medical condition cannot be fully measured by treatment costs. Our choice was a compromise, as there are no objective and comparable measures of disease burden for the entire disease landscape. Finally, state-of-the-art DTM exploits statistical inference built on term frequency when identifying the latent patterns. Therefore, high-frequency terms are likely to dominate the identified topics, which limits our capability to identify rare, yet meaningful, topics from patent documents. Furthermore, tuning the hypermeters in DTM can be more art than science.

## Appendix

Multimedia Appendix 1 The US patent classification systems and biomedicine-relevant codes (Table S1); UMLS semantic types on biomedicine (Table S2); Summary statistics of US patent data during 1995-2017 (Table S3); Multicollinearity between the relative number of patents and other factors including the relative treatment cost, the relative number of publications and the relative number of clinical trials (Table S4); Coherence scores of learned topics over different topic number for diabetes mellitus, breast cancer, and epilepsy (Figure S1).

Multimedia Appendix 2 Python script to parse patent documents in 3 different formats.

Multimedia Appendix 3 Patent coverage (Table S5), ROIs (Table S6), PHIs (Table S7), and topics (Tables S8 and S9) for over 600 diseases and medical conditions.

## Abbreviations

CPC: Cooperative Patent Classification
CUI: Concept Unique Identifier
DTM: Dynamic Topic Model
ICD-9-CM: International Classification of Diseases, Ninth Revision, Clinical Modification
ICD-10-CM: International Classification of Diseases, Tenth Revision, Clinical Modification
MeSH: Medical Subject Heading
PHI: Public Health Index
ROI: Research Opportunity Index
SGML: Standard Generalized Markup Language
UMLS: Unified Medical Language System
USPC: United States Patent Classification
