# Multimedia Appendix Correction: Technological Innovations in Disease Management: Text Mining US Patent Data From 1995 to 2017

**DOI:** 10.2196/14678

**Published:** 2019-07-05

**Authors:** Ming Huang, Maryam Zolnoori, Joyce E Balls-Berry, Tabetha A Brockman, Christi A Patten, Lixia Yao

**Affiliations:** 1 Department of Health Sciences Research Mayo Clinic Rochester, MN United States; 2 Center for Clinical and Translational Science Commuity Engagement Program Mayo Clinic Rochester, MN United States; 3 Department of Psychiatry and Psychology Mayo Clinic Rochester, MN United States

The authors of “Technological Innovations in Disease Management: Text Mining US Patent Data From 1995 to 2017” (J Med Internet Res 2019;21(4):e13316) have provided a new version of Multimedia Appendix 3.

The originally published version of Multimedia Appendix 3 contained raw treatment costs of diseases as disease burden in Table S6 that were calculated with claims data from OptumLabs. The sharing of raw data violates the data policy of OptumLabs, so those raw numbers have been removed.

The correction will appear in the online version of the paper on the JMIR website on July 5, 2019, together with the publication of this correction notice. Because this was made after submission to PubMed, PubMed Central, and other full-text repositories, the corrected article also has been resubmitted to those repositories.

## Appendix

Multimedia Appendix 3 Patent coverage (Table S5), ROIs (Table S6), PHIs (Table S7), and topics (Tables S8 and S9) for over 600 diseases and medical conditions.

