# Supplementary material for: Technological Innovations in Disease Management: Text Mining US Patent Data From 1995 to 2017
Source: J Med Internet Res. 2019 Apr 30;21(4):e13316. doi: 10.2196/13316 (PMC6611693; doi:10.2196/13316)
Supplement: Multimedia Appendix 1 [file jmir_v21i4e13316_app1.pdf]

Technological Innovations in Disease Management: Text Mining US Patent Data From 1995 to 2017

Multimedia Appendix 1

Ming Huang[1], PhD; Maryam Zolnoori[1], PhD; Joyce E Balls-Berry[1], MPE, PhD; Tabetha A Brockman[2,3], MA; Christi A Patten[2,3], PhD; and Lixia Yao[1], PhD

[1]Department of Health Sciences Research, Mayo Clinic, Rochester, Minnesota, United States

[2]Center for Clinical and Translational Science, Commuity Engagement Program, Mayo Clinic, Rochester, Minnesota, United States

[3]Department of Psychiatry and Psychology, Mayo Clinic, Rochester, Minnesota, United States

Corresponding Author:
Lixia Yao, PhD
Department of Health Sciences Research
Mayo Clinic
200 First Street SW
Rochester, MN, 55905
United States
Phone: 1 507-293-7953
Fax: 1 507-284-1516
Email: Yao.Lixia@mayo.edu

Table S1 The US patent classification systems and biomedicine-relevant codes

Classification System	Code Class	Description
USPC	128	surgery
	351	optics: eye examining, vision testing and correcting
	424	drug, bio-affecting and body treating compositions
	433	dentistry
	435	chemistry: molecular biology and microbiology
	436	chemistry: analytical and immunological testing
	514	drug, bio-affecting and body treating compositions
	600	surgery
	601	surgery: kinesitherapy
	602	surgery: splint, brace, or bandage
	604	surgery
	606	surgery
	607	surgery: light, thermal, and electrical application
	623	prosthesis (i.e., artificial body members), parts thereof, or aids and accessories therefor
	800	multicellular living organisms and unmodified parts thereof and related processes
	930	peptide or protein sequence
	D24	medical and laboratory equipment
CPC	A61B	diagnosis; surgery; identification
	A61C	dentistry; apparatus or methods for oral or dental hygiene
	A61D	veterinary instruments, implements, tools, or methods
	A61F	filters implantable into blood vessels; prostheses; devices providing patency to, or preventing collapsing of, tubular structures of the body, e.g. stents; orthopaedic, nursing or contraceptive devices; fomentation; treatment or protection of eyes or ears; bandages, dressings or absorbent pads; first-aid kits
	A61G	transport or accomodation for patients; operating tables or chairs; chairs for dentistry; funeral devices

	A61H	physical therapy apparatus, e.g. devices for locating or stimulating reflex points in the body; artificial respiration; massage; bathing devices for special therapeutic or hygienic purposes or specific parts of the body
	A61J	containers specially adapted for medical or pharmaceutical purposes; devices or methods specially adapted for bringing pharmaceutical products into particular physical or administering forms; devices for administering food or medicines orally; baby comforters; devices for receiving spittle
	A61K	preparations for medical, dental, or toilet purposes
	A61L	methods or apparatus for sterilising materials or objects in general; disinfection, sterilisation, or deodorisation of air; chemical aspects of bandages, dressings, absorbent pads, or surgical articles; materials for bandages, dressings, absorbent pads, or surgical articles
	A61M	devices for introducing media into, or onto, the body; devices for transducing body media or for taking media from the body; devices for producing or ending sleep or stupor
	A61N	electrotherapy; magnetotherapy; radiation therapy; ultrasound therapy
	A61P	specific therapeutic activity of chemical compounds or medicinal preparations
	A61Q	specific use of cosmetics or similar toilet preparations

Table S2 UMLS semantic types on biomedicine

Semantic group	Semantic type
Anatomy	Anatomical Structure
Anatomy	Body Location or Region
Anatomy	Body Part, Organ, or Organ Component
Anatomy	Body Space or Junction
Anatomy	Body Substance
Anatomy	Body System
Anatomy	Cell
Anatomy	Cell Component
Anatomy	Embryonic Structure
Anatomy	Fully Formed Anatomical Structure
Anatomy	Tissue
Chemicals & Drugs	Amino Acid, Peptide, or Protein
Chemicals & Drugs	Antibiotic
Chemicals & Drugs	Biologically Active Substance
Chemicals & Drugs	Biomedical or Dental Material
Chemicals & Drugs	Chemical
Chemicals & Drugs	Chemical Viewed Functionally
Chemicals & Drugs	Chemical Viewed Structurally
Chemicals & Drugs	Clinical Drug
Chemicals & Drugs	Element, Ion, or Isotope
Chemicals & Drugs	Enzyme
Chemicals & Drugs	Hazardous or Poisonous Substance
Chemicals & Drugs	Hormone
Chemicals & Drugs	Immunologic Factor
Chemicals & Drugs	Indicator, Reagent, or Diagnostic Aid
Chemicals & Drugs	Inorganic Chemical
Chemicals & Drugs	Nucleic Acid, Nucleoside, or Nucleotide
Chemicals & Drugs	Organic Chemical
Chemicals & Drugs	Pharmacologic Substance
Chemicals & Drugs	Receptor
Chemicals & Drugs	Vitamin
Devices	Drug Delivery Device
Devices	Medical Device
Devices	Research Device
Disorders	Acquired Abnormality
Disorders	Anatomical Abnormality
Disorders	Cell or Molecular Dysfunction

Disorders	Congenital Abnormality
Disorders	Disease or Syndrome
Disorders	Experimental Model of Disease
Disorders	Finding
Disorders	Injury or Poisoning
Disorders	Mental or Behavioral Dysfunction
Disorders	Neoplastic Process
Disorders	Pathologic Function
Disorders	Sign or Symptom
Genes & Molecular Sequences	Amino Acid Sequence
Genes & Molecular Sequences	Carbohydrate Sequence
Genes & Molecular Sequences	Gene or Genome
Genes & Molecular Sequences	Molecular Sequence
Genes & Molecular Sequences	Nucleotide Sequence
Living Beings	Bacterium
Living Beings	Patient or Disabled Group
Living Beings	Population Group
Living Beings	Professional or Occupational Group
Living Beings	Virus
Objects	Manufactured Object
Phenomena	Biologic Function
Phenomena	Laboratory or Test Result
Physiology	Cell Function
Physiology	Clinical Attribute
Physiology	Genetic Function
Physiology	Mental Process
Physiology	Molecular Function
Physiology	Organism Attribute
Physiology	Organism Function
Physiology	Organ or Tissue Function
Physiology	Physiologic Function
Procedures	Diagnostic Procedure
Procedures	Health Care Activity
Procedures	Laboratory Procedure
Procedures	Molecular Biology Research Technique
Procedures	Research Activity
Procedures	Therapeutic or Preventive Procedure

Table S3 Summary statistics of US patent data during 1995-2017

Year	Number of patents*	Number of patents related to biomedicine	Percentage of patents related to biomedicine	Number of Mapped Root PheCodes
1995	113,955	13,635	11.97	502
1996	121,816	15,347	12.60	525
1997	124,192	18,070	14.55	531
1998	163,268	23,314	14.28	546
1999	169,262	23,629	13.96	559
2000	176,198	22,716	12.89	563
2001	184,211	24,078	13.07	575
2002	184,494	23,687	12.84	569
2003	187,104	23,906	12.78	567
2004	181,413	19,349	10.67	566
2005	160,989	16,322	10.14	540
2006	200,969	20,165	10.03	563
2007	187,510	19,588	10.45	554
2008	189,558	18,172	9.59	544
2009	196,687	20,068	10.20	572
2010	250,550	28,961	11.56	572
2011	254,066	30,267	11.91	580
2012	283,405	34,885	12.31	595
2013	310,279	39,667	12.78	594
2014	334,110	43,124	12.91	592
2015	334,128	39,065	11.69	599
2016	341,954	36,807	10.76	594
2017	360,211	39,795	11.05	596

* Patent documents are downloaded from USPTO Bulk Data Storage System (https://bulkdata.uspto.gov/).

ROI and PHI Analysis

Table S4 Multicollinearity between the relative number of patents and other factors including the relative treatment cost, the relative number of publications and the relative number of clinical trials

Factors	Variance Inflation Factor[1]
Treatment cost	1.13
Publication	2.43
Clinical Trial	2.52

Topic modeling

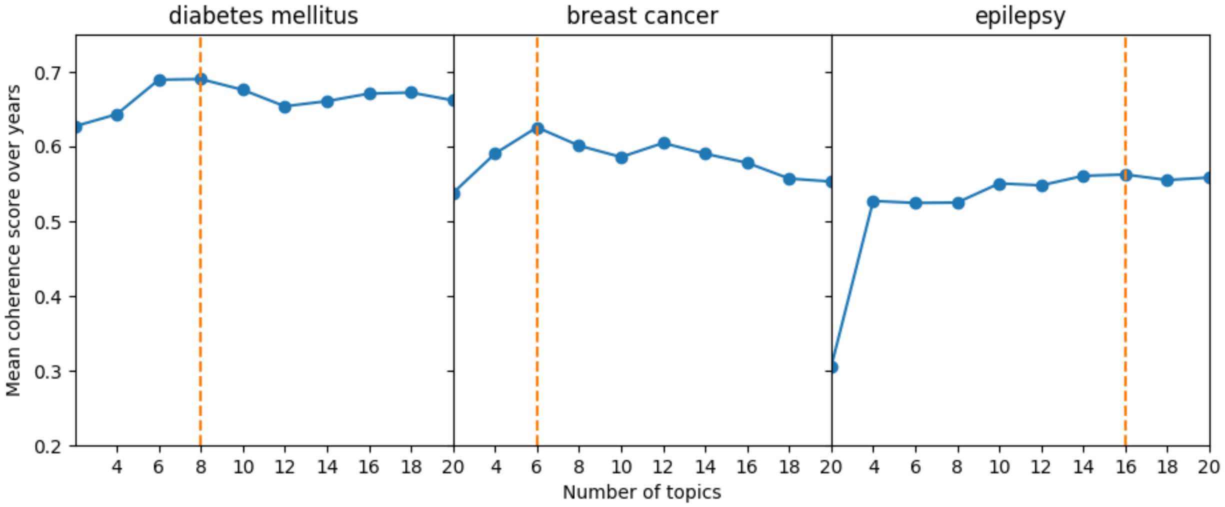

Figure S1 Coherence scores of learned topics over different topic number for diabetes mellitus, breast cancer, and epilepsy

Reference

1 James, G., Witten, D., Hastie, T. & Tibshirani, R. *An introduction to statistical learning*. Vol. 112 (Springer, 2013).
